# Establishing immune scoring model based on combination of the number, function, and phenotype of lymphocytes

**DOI:** 10.18632/aging.103208

**Published:** 2020-05-12

**Authors:** Guoxing Tang, Xu Yuan, Ying Luo, Qun Lin, Zhishui Chen, Xue Xing, Huijuan Song, Shiji Wu, Hongyan Hou, Jing Yu, Liyan Mao, Weiyong Liu, Feng Wang, Ziyong Sun

**Affiliations:** 1 Department of Laboratory Medicine, Tongji Hospital, Tongji Medical College, Huazhong University of Science and Technology, Wuhan, China; 2 Institute of Organ Transplantation, Tongji Hospital, Tongji Medical College, Huazhong University of Science and Technology, Wuhan, China; 3 Key Laboratory of Organ Transplantation, Ministry of Education, Ministry of Public Health, Chinese Academy of Medical Sciences, Beijing, China; 4 Department of Nephrology, Tongji Hospital, Tongji Medical College, Huazhong University of Science and Technology, Wuhan, China; 5 The Third Affiliated Hospital of Zhengzhou University, Zhengzhou, China; 6 Center for Cellular and Molecular Diagnosis, Biochemistry and Molecular Biology, Tulane University School of Medicine, New Orleans, LA 70112, USA

**Keywords:** immune scoring model, lymphocyte number, lymphocyte function, lymphocyte phenotype, host immunity

## Abstract

Background: Quantitatively assessing host immunity remains a challenge in clinical practice.

Results: Most parameters in lymphocyte number, function and phenotype were correlated with age. The reference ranges of these parameters were established in four age groups (children, adolescents, adults, and elders). The numbers of CD4^+^ T cells, CD8^+^ T cells, B cells, but not NK cells, were negatively correlated with age. However, the function of CD4^+^ T cells, CD8^+^ T cells and NK cells was positively correlated with age. The expression of CD28 on T cells gradually decreased with increasing age and was negatively correlated with their function. An opposite phenomenon was observed in the expressions of HLA-DR and CD45RO on T cells. An immune scoring model was established by using 8 parameters (CD4^+^ T cell number × function, CD28^+^CD4^+^ T cell number, HLA-DR^+^CD4^+^ T cell number, CD45RO^+^CD4^+^ T cell number, CD8^+^ T cell number × function, CD28^+^CD8^+^ T cell number, HLA-DR^+^CD8^+^ T cell number, NK cell number × function) from the results of lymphocyte number, function, and phenotype. This immune scoring model showed sensitivities of 70% and 71.4% in determining hyper-immune and hypo-immune status, respectively.

Conclusions: An immune scoring model based on combination of lymphocyte number, function, and phenotype shows potential value in quantitatively assessing host immunity.

Methods: 261 healthy individuals aged 1 to 82 years were recruited from Tongji Hospital. The number, function, and phenotype of CD4^+^ T cells, CD8^+^ T cells and NK cells were simultaneously determined.

## INTRODUCTION

The immune system plays a crucial role in maintaining body health, not only by protecting the host against pathogenic agents including bacteria, fungi and virus but also by eliminating aged, mutant or dead cells in the body [1–3]. The abnormal immune function can cause many hazards, such as infectious disease, cancer, and autoimmune disease. Nevertheless, the most critical issue is the lack of laboratory tests that can quantitatively detect immune status. Currently, the clinicians commonly judge the immune status of hosts according to whether patients have underlying diseases such as diabetes mellitus, malignancy and chronic renal failure, and this is obviously inaccurate. Thus, the development of rapid and accurate methods for the detection and quantification of host immunity is of increasing importance in clinical practice.

Lymphocytes, which mainly consist of T cells, B cells and NK cells, are the key effector cells of immune system. Meanwhile, lymphocytes regulate immune system via activation, cytotoxicity, and secretion of cytokines. There are many methods reported to detect lymphocyte function in scientific research. [^3^H]-thymidine incorporation and carboxyfluorescein diacetate succinimidyl ester (CFSE)-labeling assay are used to determine the proliferation of lymphocytes [4–6]. The activation markers including CD25, CD69 and HLA-DR are measured to reflect the activation of T cells and NK cells [7, 8]. Chromium (^51^Cr)-release assay has the ability to detect the cytotoxicity of CD8^+^ T cells and NK cells [9]. We also used CFSE/PI-labeled target cells to determine the cytotoxicity of NK cells [9, 10]. However, although these methods are widely used in previous studies, most of them are not suitable for clinical application due to radiation hazards, too complicated and time-consuming administration. The activation markers are easily detected in clinical laboratory, but they cannot represent lymphocyte function comprehensively.

Our previous study has shown that interferon-gamma (IFN-γ) production of CD4^+^ and CD8^+^ T cells and NK cells after 4 hours of phorbol-12-myristate-13-acetate/Ionomycin (PMA/Ionomycin) stimulation was positively correlated with the activation, chemotaxis, and cytotoxicity of them, which suggests that IFN-γ producing capability can be used as a marker of lymphocyte function [11]. This method is simple, rapid, and safe and has great value in clinical application. Recently, although many studies have focused on the change of lymphocyte number, function and phenotype in healthy individuals with different age groups or in patients with different diseases, few have investigated these aspects simultaneously [11–13]. For one thing, neither the number nor the phenotype can represent the function of lymphocytes, and for another, individuals with enhanced lymphocyte function may be in immunosuppressive state due to reduced lymphocyte number. Thus, understanding the host's immune status depends on comprehensive analysis of the number, function, and phenotype of lymphocytes.

In this study, based on our previous established PMA/ionomycin-stimulated lymphocyte function assay, we investigated the number, function, and phenotype of CD4^+^ T cells, CD8^+^ T cells and NK cells simultaneously in healthy individuals in different age and gender groups. We found that the number, function and phenotype of lymphocytes showed significant correlation with each other. We first established an immune scoring model based on combination of lymphocyte number, function and phenotype, and this model showed potential value in determining hyper-immune or hypo-immune status.

## RESULTS

### Participants’ characteristics

A total of 261 healthy individuals fulfilled the inclusion criteria were recruited for the study, including 168 (64.4%) males and 93 (35.6%) females. The median age of the healthy individuals was 34 years (range: 1-82 years). The healthy individuals were divided into four groups according to their age range: 47 (18.01%) individuals (35 males, 12 females) aged 1-5 years were classified as children; 72 (27.59%) individuals (52 males, 20 females) aged 6-18 years were classified as adolescents; 90 (34.48%) individuals (53 males, 37 females) aged 18-65 years were classified as adults; 52 (19.92%) individuals (28 males, 24 females) aged >65 years were classified as elders.

### The reference ranges of lymphocyte number, function and phenotype in different age and gender groups

**Figure 1:**
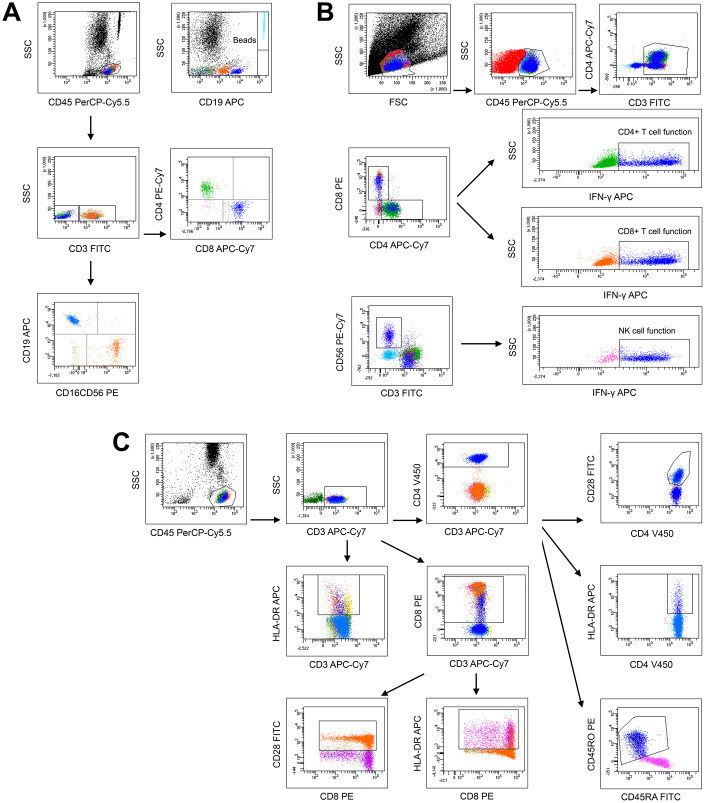
**The analysis templates of flow cytometry** for lymphocyte number (**A**), function (**B**), and phenotype (**C**).

The analysis templates of flow cytometry for lymphocyte number, function and phenotype are shown in Figure 1A–1C, respectively. Given that all parameters in lymphocyte number, function and phenotype were correlated with age, the reference ranges of these parameters were established in four age groups (children, adolescents, adults, and elder). Four parameters were used to represent lymphocyte number: CD4^+^ T cell number, CD8^+^ T cell number, B cell number, and NK cell number. Three parameters were used to represent lymphocyte function: CD4^+^ T cell function (IFN-γ^+^CD4^+^ T cells %), CD8^+^ T cell function (IFN-γ^+^CD8^+^ T cells %), and NK cell function (IFN-γ^+^ NK cells %). Five parameters were used to represent lymphocyte phenotype: CD28^+^CD4^+^ T cells %, HLA-DR^+^CD4^+^ T cells %, CD45RO^+^CD4^+^ T cells %, CD28^+^CD8^+^ T cells %, and HLA-DR^+^CD8^+^ T cells %. The reference ranges of these parameters in healthy individuals in different age groups are shown in Table 1. The original TBNK percentage results are shown in Supplementary Table 1. Moreover, most parameters in lymphocyte number, function, and phenotype had no significant difference between different gender groups. The reference ranges of these parameters in different gender groups are shown in Supplementary Table 2.

**Table 1:** Reference ranges of lymphocyte number, function, and phenotype in different age groups.

**Parameters**		**All**	**Children**	**Adolescents**	**Adults**	**Elders**	**p**
		**N=261**	**N=47**	**N=72**	**N=90**	**N=52**	
Age	Mean±SD (Range)	33.68±26.63 (1-82)	3.81±1.04 (1-5)	10.81±3.74 (6-18)	46±13.79 (18-65)	71.04±4.39 (66-82)	
Sex	Male:Female	168:93	35:12	52:20	53:37	28:24	
**Number**							
CD4^+^ T cell number (/ml)	Mean±SD (2.5%-97.5%)	836±355 (374-1881)	1260±399 (635-1979)	927±287 (560-1653)	648±203 (360-1074)	652±187 (367-1007)	<0.001
CD8^+^T cell number (/ml)	Mean±SD (2.5%-97.5%)	576±310 (154-1459)	880±343 (426-1553)	728±262 (397-1382)	424±171 (180-847)	355±154 (116-681)	<0.001
B cell number (/ml)	Mean±SD (2.5%-97.5%)	358±252 (73-1006)	695±251 (251-1240)	457±173 (218-905)	184±81 (53-352)	216±140 (67-537)	<0.001
NK cell number (/ml)	Mean±SD (2.5%-97.5%)	383±219 (103-920)	381±243 (89-905)	345±228 (86-934)	364±165 (154-732)	473±239 (125-1000)	<0.05
**Function**							
IFN-g^+^CD4^+^ T cells (%)	Mean±SD (2.5%-97.5%)	17.84±8.85 (6.62-36.81)	10.23±4.16 (4.08-17.17)	12.28±4.86 (5.43-20.35)	23.72±8.12 (12.34-40.53)	22.26±7.52 (8.83-34.43)	<0.001
IFN-g^+^CD8^+^ T cells (%)	Mean±SD (2.5%-97.5%)	46.25±22.43 (13.59-87.72)	26.89±10.77 (8.95-46.18)	29.13±10.83 (13.63-56.57)	56.08±17.92 (22.76-87.38)	70.46±14.33 (42.84-92.36)	<0.001
IFN-g^+^ NK cells (%)	Mean±SD (2.5%-97.5%)	72.68±12.65 (43.88-90.94)	67.28±14.57 (40.76-85.99)	67.77±13.16 (39.70-87.66)	77.29±9.95 (57.73-91.48)	76.37±9.52 (59.79-90.61)	<0.001
**Phenotype**							
CD28^+^CD4^+^ T cells (%)	Mean±SD (2.5%-97.5%)	94.95±7.03 (73.33-99.97)	98.17±4.03 (89.50-99.98)	97.92±2.72 (88.95-99.95)	92.81±7.6 (71.81-99.88)	91.61±9.10 (66.26-99.83)	<0.001
HLA-DR^+^CD4^+^ T cells (%)	Mean±SD (2.5%-97.5%)	14.33±7.45 (5.42-32.78)	9.68±4.79 (5.29-24.40)	10.64±4.15 (5.11-19.71)	16.23±7.02 (5.97-34.34)	20.35±8.25 (9.07-40.22)	<0.001
CD45RO+CD4^+^T cells (%)	Mean±SD(2.5%-97.5%)	50.89±18.88 (20.58-88.47)	28.77±7.71 (13.63-43.39)	40.37±9.88 (22.48-58.08)	61.09±14 (36.21-88.06)	67.82±14.07 (37.46-93.85)	<0.001
CD28^+^CD8^+^ T cells (%)	Mean±SD (2.5%-97.5%)	62.06±17.3 (26.41-88.91)	71.58±12.49 (52.47-91.71)	71.39±12.36 (45.28-88.65)	58±15.87 (26.72-84.13)	47.57±16.5 (20.60-82.40)	<0.001
HLA-DR^+^CD8^+^ T cells (%)	Mean±SD (2.5%-97.5%)	34.93±17.12 (9.97-71.53)	23.26±11.82 (7.42-47.14)	25.13±10.45 (9.98-46.31)	39.09±15.71 (14.96-72.95)	51.83±13.98 (22.97-74.98)	<0.001

SD: standard deviation. *P* means association between different parameters and age in all participants by using Spearman's rank correlation test.

Some parameters which can reflect the combination effect between lymphocyte number and function or between lymphocyte number and phenotype (CD4^+^ T cell number × function, CD4^+^ T cell number × CD28^+^CD4^+^ T cells %, CD4^+^ T cell number × HLA-DR^+^CD4^+^ T cells %, CD4^+^ T cell number × CD45RO^+^CD4^+^ T cells %, CD8^+^ T cell number × function, CD8^+^ T cell number × CD28^+^CD8^+^ T cells %, CD8^+^ T cell number × HLA-DR^+^CD8^+^ T cells %, NK cell number × function) were further calculated, and the reference ranges of these calculated parameters are shown in Table 2.

**Table 2:** Reference ranges of calculated parameters in different age groups.

**Parameters**		**All**	**Children**	**Adolescents**	**Adults**	**Elders**	**p**
		**N=261**	**N=47**	**N=72**	**N=90**	**N=52**	
Age	Mean±SD (Range)	33.68±26.63 (1-82)	3.81±1.04 (1-5)	10.81±3.74 (6-18)	46±13.79 (18-65)	71.04±4.39 (66-82)	
CD4^+^ T cell number × function (/ml)	Mean±SD (2.5%-97.5%)	134±66 (56-293)	125±58 (57-225)	108±40 (57-209)	153±75 (61-328)	147±73 (44-311)	<0.001
CD8^+^ T cell number × function (/ml)	Mean±SD (2.5%-97.5%)	237±139 (72-567)	240±143 (65-565)	212±126 (86-489)	242±143 (79-564)	259±74 (67-553)	<0.05
NK cell number × function (/ml)	Mean±SD (2.5%-97.5%)	283±175 (49-736)	254±157 (37-585)	241±184 (44-780)	286±142 (90-674)	216±125 (80-832)	<0.001
CD4^+^ T cell number × CD28^+^CD4^+^ T cells % (/ml)	Mean±SD (2.5%-97.5%)	798±359 (333-1844)	1236±392 (631-1925)	909±288 (536-1638)	600±190 (318-1001)	594±173 (320-894)	<0.001
CD4^+^ T cell number × HLA-DR^+^CD4^+^ T cells % (/ml)	Mean±SD (2.5%-97.5%)	110±60 (36-257)	121±70 (43-299)	95±37 (44-176)	103±52 (35-232)	135±78 (47-339)	<0.01
CD4^+^ T cell number × CD45RO^+^CD4^+^ T cells % (/ml)	Mean±SD (2.5%-97.5%)	385±140 (192-709)	347±109 (220-557)	358±94 (225-570)	393±153 (165-735)	444±168 (219-748)	<0.001
CD8^+^ T cell number × CD28^+^CD8^+^ T cells % (/ml)	Mean±SD (2.5%-97.5%)	364±232 (82-949)	618±233 (243-1011)	508±174 (262-950)	236±98 (104-435)	134±50 (57-286)	<0.001
CD8^+^ T cell number × HLA-DR^+^CD8^+^ T cells % (/ml)	Mean±SD (2.5%-97.5%)	187±129 (41-547)	221±183 (38-565)	185±113 (67-452)	169±110 (39-478)	132±48 (46-436)	>0.05

SD: standard deviation. *P* means association between different parameters and age in all participants by using Spearman's rank correlation test.

### Correlation analysis between different lymphocyte parameters and age

**Figure 2:**
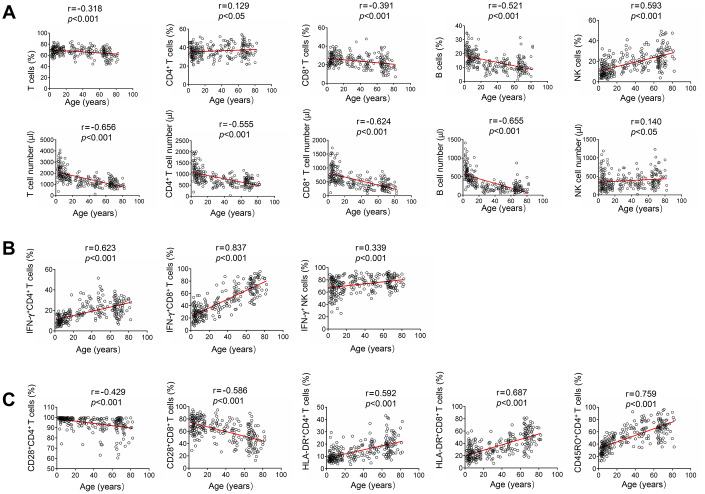
**Correlation analysis between different lymphocyte parameters and age.** (**A**) Heparinized peripheral blood was collected from study participants. The percentages and absolute numbers of CD4^+^ T cells, CD8^+^ T cells, B cells and NK cells were determined by flow cytometry. Correlation between lymphocyte count (including percentage and absolute number) and age. (**B**) PMA/ionomycin-stimulated lymphocyte function assay was performed in study participants. Correlation between lymphocyte function (including CD4^+^ T cells, CD8^+^ T cells, and NK cells) and age. (**C**) The expression of phenotype markers CD28, HLA-DR, and CD45RO on CD4^+^ and CD8^+^ T cells was analyzed by flow cytometry. Correlation between these phenotype markers and age. Each symbol represents an individual donor.

For lymphocyte number, the absolute numbers of total T cells, CD4^+^ T cells, CD8^+^ T cells and B cells were all negatively correlated with age. In contrast, both the percentage and absolute number of NK cells were positively correlated with age (Figure 2A). For lymphocyte function, the function of CD4^+^ and CD8^+^ T cells was low after birth, but increased with increasing age. Therefore, the function of both CD4^+^ and CD8^+^ T cells was positively correlated with age. The function of NK cells was also positively correlated with age. Differently, the function of NK cells was maintained at a high level after birth, and then slowly increased with increasing age (Figure 2B). For lymphocyte phenotype, the expression of naive marker CD28 on both CD4^+^ and CD8^+^ T cells was negatively correlated with age. In contrast, the expression of activated marker HLA-DR on them was positively correlated with age. The expression of memory marker CD45RO on CD4^+^ T cells was also positively correlated with age (Figure 2C).

### Correlation analysis among lymphocyte number, function and phenotype

**Figure 3:**
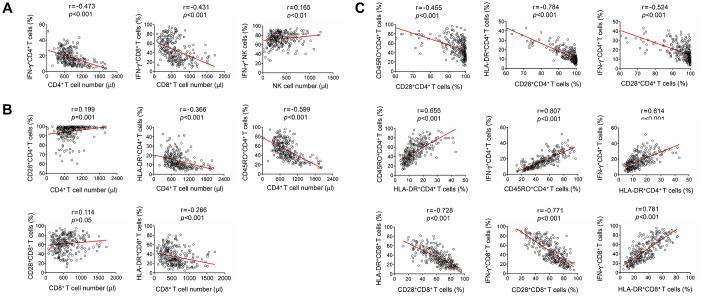
**Correlation analysis among lymphocyte number, function and phenotype.** (**A**) Correlation between lymphocyte function (including CD4^+^ T cells, CD8^+^ T cells, and NK cells) and lymphocyte number. (**B**) Correlation between lymphocyte phenotype (including the expression of CD28, HLA-DR, and CD45RO on CD4^+^ T cells or CD8^+^ T cells) and lymphocyte number. (**C**) Correlation among different lymphocyte phenotype markers (CD28, HLA-DR, and CD45RO), or correlation between lymphocyte function (including CD4^+^ and CD8^+^ T cells) and lymphocyte phenotype. Each symbol represents an individual donor.

The numbers of CD4^+^ and CD8^+^ T cells were negatively correlated with the function of them. In contrast, NK cell number was slightly positively correlated with its function (Figure 3A). The expression of CD28 on CD4^+^ T cells was positively correlated with the number of CD4^+^ T cells. Contrastingly, the expression of both HLA-DR and CD45RO on CD4^+^ T cells was negatively correlated with CD4^+^ T cell number. HLA-DR expression on CD8^+^ T cells was also negatively correlated with their number (Figure 3B). Furthermore, CD28 expression on CD4^+^ and CD8^+^ T cells was negatively correlated with HLA-DR expression on them. CD28 expression on CD4^+^ T cells was also negatively correlated with their CD45RO expression. On the other hand, CD28 expression on both CD4^+^ and CD8^+^ T cells was negatively correlated with their function. On the contrary, HLA-DR and CD45RO expression on CD4^+^ and CD8^+^ T cells was positively correlated with their function (Figure 3C).

### Establishment of immune scoring model based on combination of the number, function and phenotype of lymphocytes

**Figure 4:**
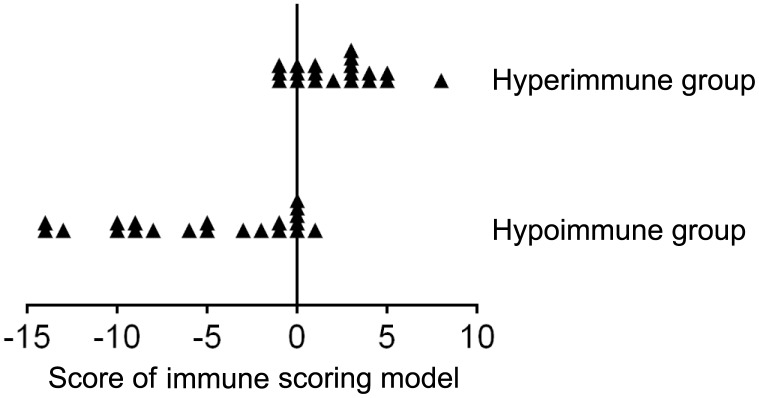
**The score distribution of the immune scoring model (based on combination of lymphocyte number, function, and phenotype) in patients with hyperimmune and hypoimmune status.**

To quantitatively evaluate host immunity, an immune scoring model based on combination of lymphocyte number, function and phenotype was established as described in method section. Twenty and twenty-one samples of peripheral blood were collected from hyperimmune and hypoimmune patients, respectively. The demographic and clinical characteristics of these patients are shown in Supplementary Table 3. To match the patients with respect to age and sex, a total of 118 healthy individuals aged 23–76 years were selected. Half of them were finally selected by using the random under-sampling method. The mean score of the immune scoring model in these 59 healthy individuals is 0. In hyperimmune group, the score of the model ranged from -1 to 7, and the mean score was 2.15 (score < 0, n = 3; score = 0, n = 3; score > 0, n = 14). In hypoimmune group, the score of the model ranged from -14 to 1, the mean score was -5.19 (score < 0, n = 15; score = 0, n=5; score > 0, n = 1). If using score > 0 as cutoff value, the immune scoring model showed a sensitivity of 70% and a specificity of 100% in determining hyperimmune status. If using score < 0 as cutoff value, the immune scoring model showed a sensitivity of 71.4% and a specificity of 100% in determining hypoimmune status. The score distribution of the participants in different immune status is shown in Figure 4.

## DISCUSSION

The immune system plays a crucial role in maintaining health. However, how to quantitatively assess host immunity is still a challenge in clinical practice. Clinicians commonly use clinical information combined with a few laboratory tests such as peripheral white blood cell count and TBNK lymphocyte count to determine host immunity, which is obviously inaccurate as lymphocyte number cannot represent lymphocyte function. Based on our previously established PMA/ionomycin-stimulated lymphocyte function assay, we simultaneously assessed the number, function and phenotype of lymphocytes in this study. A further established immune scoring model based on combination of these indicators showed good performance in quantitatively determining host immunity.

In accordance with previous studies, our results demonstrated that the number of both T cells (including CD4^+^ and CD8^+^ T cells) and B cells gradually decreased with increasing age [12, 13]. Previous studies have also shown that the diversity of B cell phenotype is decreased in elderly people, which results in decreased protective effect of vaccination in them compared with young people [12, 14–16]. These data suggest that the number of adaptive immune cells gradually decreases during life. Furthermore, NK cells are one of the key components of the innate immune system. Contrastingly, however, the number of NK cells slowly increases with increasing age. This is different from previous studies showing that NK cell count maintains stable level in elderly individuals [11, 12]. One of the possible reasons is that participants in a continuous age range between 1 and 82 years were used to describe age-related change in this study, whereas previous studies only included a limited age group to determine the number of NK cells. Our data suggest that age-related changes between the number of adaptive and innate immune cells are different.

Rare studies have determined the relationship between lymphocyte function and age. The most probable cause is that current methods, such as CFSE proliferation assay and CD107a degranulation assay, are complicated and time-consuming and not suitable for clinical application [5, 17–19]. Based on our previously established PMA/ionomycin-stimulated lymphocyte function assay, we found a robust positive correlation between T cell function and age [11]. We proposed that the increase of function in CD4^+^ and CD8^+^ T cells with increasing age is to maintain certain degree of immune function, as the numbers of CD4^+^ and CD8^+^ T cells have been declining during life. Interestingly, the function of NK cells was maintained at a high level after birth and slowly increased with increasing age. These data suggest that innate immunity is more important for children and elders, because the function of adaptive immune cells is immature in children and the number of them is insufficient in elders. These data are consistent with previous findings indicating that NK cells play an important role in the immunity of elders and may be interpreted as a factor of longevity [20–23].

Many surface molecules are selectively expressed on different lymphocyte subsets and are described as lymphocyte phenotype. We chose several classical phenotype markers of lymphocytes including CD28 (naive marker), CD45RA (naive marker), HLA-DR (activated marker) and CD45RO (memory marker) to reflect host immunity. These phenotype markers are correlated with lymphocyte function and are known to play an important role in many diseases such as infection, tumor, and autoimmune disease [24–27]. As expected, our data demonstrated that the expression of CD28 on T cells gradually decreased with increasing age and was negatively correlated with their function. An opposite phenomenon was observed in the expressions of HLA-DR and CD45RO on T cells. These data suggest that the potential of immunity in elders is reduced and that a high expression of CD28 can be used to predict longevity, which is in accordance with previous studies [28, 29]. Since CD45RA and CD45RO are different CD45 isoforms expressed on T cells, we did not analyze the results of CD45RA in case of repetition.

Both the number and function of lymphocytes are critical to maintain normal immunity. Either the decreased lymphocyte number or the impaired lymphocyte function can lead to immunodeficiency. Thus, the assessment of immunity depends on the combination of lymphocyte number and function. Nevertheless, rare studies have established immune scoring model to assess immunity based on combination of these two aspects. Since different lymphocyte phenotypes are also correlated with different lymphocyte function, we first established an immune scoring model based on combination of the number, function and phenotype of lymphocytes to comprehensively assess host immunity. The imbalance of immunity leads to many immune-related diseases, such as autoimmune disease (hyperimmune status) [30] and opportunistic infection (hypoimmune status) [31–36]. The validation data from patients with different immune status showed that the model had high sensitivities in the determination of both hyperimmune and hypoimmune status. An immune scoring model based on combination of lymphocyte number and function was also established. The model had a lower sensitivity than above model (Supplementary Figure 1)

Our study proposes a quantitative model for the evaluation of host immunity by combining the number, function and phenotype of lymphocytes. Previous studies have also incorporated relevant factors of immune system for differential diagnosis of diseases [37–39]. Castelblanco et al used a model by combination of adiponectin, soluble tumor necrosis factor-α receptor 2, interleukin-6, hs-CRP and leukocyte number to differentiate among different types of diabetes [37]. Qiu et al used neutrophil–lymphocyte ratio (NLR) to evaluate triple-negative breast cancer. Patients with NLR lower than 2.85 exhibited significantly higher overall survival and disease-free survival than those with higher NLR [38]. Qiu et al compared the differences in lymphocyte subsets between cancer patients and healthy people [39]. Although many studies compared the number and phenotype of lymphocytes in patients with different diseases, rare studies further established a model.

The immune scoring model established in the present study can assist in the diagnosis and prognosis of various diseases. We speculated that the model can be also used to monitor the effect of treatment in immune-related diseases. For example, the model can be used to comprehensively evaluate the host’s immune status and determine the optimal dosage of immunosuppressant in patients after transplantation. This model can also assist clinicians to determine the causes of disease and choose the right treatment in patients with infectious diseases.

Taken together, we put forward that an immune scoring model based on combination of lymphocyte number, function and phenotype has potential value in the assessment of host immunity.

## MATERIALS AND METHODS

### Subjects

This study was carried out from May 2018 to June 2019 at Tongji Hospital (the largest hospital in central China). A total of 261 healthy individuals (168 males, 93 females) aged 1-82 years were recruited. These subjects were determined by clinical interview and physical examination to be free of illness. Exclusion criteria were as follows: pregnancy, atherosclerosis and vascular disease, cardiopathy, chronic nephropathy, hepatobiliary disease, allergic disease, autoimmune disease, hematological disease, myopathy, burns and muscle trauma, positive for HIV, HBV, HCV, CMV, and syphilis antibodies. Another two groups of patients with hyper-immune and hypo-immune status were also recruited. Patients with autoimmune diseases were defined according to the criteria of the American College of Rheumatology and were classified as hyperimmune group. Patients infected with opportunistic pathogens including aspergillus, pneumocystis carinii and cryptococcus neoformans were classified as hypoimmune group. This study was approved by the ethical committee of Tongji Hospital, Tongji Medical College, Huazhong University of Science and Technology, China.

### Lymphocyte count

Heparinized peripheral blood was collected from study participants. The percentages and absolute numbers of CD4^+^ T cells, CD8^+^ T cells, B cells and NK cells were determined by using TruCOUNT tubes and BD Multitest 6-color TBNK Reagent Kit (BD Biosciences) according to the manufacturer's instructions. In brief, 50 μl of whole blood was labeled with 6-color TBNK antibody cocktail for 15 min in room temperature. After adding 450 μl of FACS Lysing Solution, samples were analyzed with FACSCanto flow cytometer using FACSCanto clinical software (BD Biosciences). Cells with CD45 high expression and with low side scatter were gated as lymphocytes. TruCOUNT beads were gated based on side scatter and fluorescence intensity. CD3^+^ cells in lymphocyte gate were defined as total T cells. CD4^+^CD8^-^ and CD8^+^CD4^-^ cells in CD3^+^ cells were defined as CD4^+^ T cells and CD8^+^ T cells, respectively. CD19^+^ and CD16^+^CD56^+^ cells in CD3^-^ cells were defined as B cells and NK cells, respectively.

### Lymphocyte function analysis

Heparinized peripheral blood was collected from study participants. PMA/ionomycin-stimulated lymphocyte function assay was performed as described previously [11]. The procedures are described in brief as follows: 1) 100 μl of whole blood was diluted with 400 μl of IMDM medium; 2) the diluted whole blood was incubated in the presence of Leukocyte Activation Cocktail (Becton Dickinson GolgiPlug) for 4 h; 3) the cells were labeled with monoclonal antibodies (anti-CD45, anti-CD3, anti-CD4, anti-CD56, and anti-CD8); 4) the cell were fixed and permeabilized; 5) the cells were stained with intracellular anti-IFN-γ antibody; and 6) the cells were analyzed with FACSCanto flow cytometer. The percentages of IFN-γ^+^ cells in different cell subsets were defined as the function of them (e.g., the percentage of IFN-γ^+^ cells in CD3^+^CD4^+^CD8^-^ cells was defined as the function of CD4^+^ T cells; the percentage of IFN-γ^+^ cells in CD3^+^CD8^+^CD4^-^ cells was defined as the function of CD8^+^ T cells; the percentage of IFN-γ^+^ cells in CD3^-^CD56^+^ cells was defined as the function of NK cells).

### Lymphocyte phenotype analysis

Heparinized peripheral blood was collected from study participants. The following monoclonal antibodies were added to 100 μl of whole blood: anti-CD45, anti-CD3, anti-CD4, anti-CD8, anti-CD28, anti-HLA-DR, anti-CD45RA, and anti-CD45RO (BD Biosciences). Isotype controls with irrelevant specificities were included as negative controls. All of these cell suspensions were incubated for 20 min at room temperature. After lysing red blood cells, the cells were washed and resuspended in 200 μl of PBS. The cells were then analyzed with FACSCanto flow cytometer.

### Establishment of immune scoring model

The following 8 parameters were used for establishing immune scoring model: CD4^+^ T cell number × function, CD28^+^CD4^+^ T cell number, HLA-DR^+^CD4^+^ T cell number, CD45RO^+^CD4^+^ T cell number, CD8^+^ T cell number × function, CD28^+^CD8^+^ T cell number, HLA-DR^+^CD8^+^ T cell number, and NK cell number × function. The reference range of each parameter was established in healthy individuals according to the above-described methods. The immune scoring model which is similar with Sequential Organ Failure Assessment scoring in sepsis was established [40, 41], as the following rules: 1) if the values of these parameters were higher than 1.5 times the upper limit of the normal reference range, score of + 2 was recorded; 2) if the values of these parameters were between 1 and 1.5 times the upper limit of the normal reference range, score of + 1 was recorded; 3) if the values of these parameters were within the normal reference range, score of 0 was recorded; 4) if the values of these parameters were between 0.5 and 1 times the lower limit of the normal reference range, score of -1 was recorded; and 5) if the values of these parameters were lower than 0.5 times the lower limit of the normal reference range, score of -2 was recorded. The scores of these 8 parameters for each individual were summarized to calculate the total score.

### Statistical analysis

Statistical significance between different groups of participants was analyzed using the Mann–Whitney *U*-test. Spearman's rank correlation test for non-parametric data was employed to analyze the relationship between two factors. The reference ranges of parameters were determined by using the 2.5–97.5 percentile nonparametric range. The statistical analysis was performed using GraphPad Prism version 6 (GraphPad Software, San Diego, CA, USA). Statistical significance was determined as *p* < 0.05 (*p < 0.05, **p < 0.01, ***p < 0.001).

## Supplementary Material

Supplementary Figure 1

Supplementary Tables

## References

[1] Salazar N, Arboleya S, Fernández-Navarro T, de Los Reyes-Gavilán CG, Gonzalez S, Gueimonde M. Age-Associated Changes in Gut Microbiota and Dietary Components Related with the Immune System in Adulthood and Old Age: A Cross-Sectional Study. Nutrients. 2019; 11:E1765. 10.3390/nu11081765 31370376 PMC6722604

[2] Brestoff JR, Artis D. Commensal bacteria at the interface of host metabolism and the immune system. Nat Immunol. 2013; 14:676–84. 10.1038/ni.2640 23778795 PMC4013146

[3] Amankulor NM, Kim Y, Arora S, Kargl J, Szulzewsky F, Hanke M, Margineantu DH, Rao A, Bolouri H, Delrow J, Hockenbery D, Houghton AM, Holland EC. Mutant IDH1 regulates the tumor-associated immune system in gliomas. Genes Dev. 2017; 31:774–86. 10.1101/gad.294991.116 28465358 PMC5435890

[4] Hross S, Hasenauer J. Analysis of CFSE time-series data using division-, age- and label-structured population models. Bioinformatics. 2016; 32:2321–29. 10.1093/bioinformatics/btw131 27153577

[5] Lašťovička J, Rataj M, Bartůňková J. Assessment of lymphocyte proliferation for diagnostic purpose: comparison of CFSE staining, Ki-67 expression and ^3^H-thymidine incorporation. Hum Immunol. 2016; 77:1215–22. 10.1016/j.humimm.2016.08.012 27562802

[6] Bocharov G, Luzyanina T, Cupovic J, Ludewig B. Asymmetry of Cell Division in CFSE-Based Lymphocyte Proliferation Analysis. Front Immunol. 2013; 4:264. 10.3389/fimmu.2013.00264 24032033 PMC3759284

[7] Guo Q, Zhang Z, Zhao P, Zou S, Li L, Li N, Sun W, Wei X, Hou L, Yang Z, Gao D. Bispecific antibody activated T cells: A newly developed T cells with enhanced proliferation ability and cytotoxicity. Immunol Lett. 2020; 220:79–87. 10.1016/j.imlet.2019.12.010 31901377

[8] Cibrián D, Sánchez-Madrid F. CD69: from activation marker to metabolic gatekeeper. Eur J Immunol. 2017; 47:946–53. 10.1002/eji.201646837 28475283 PMC6485631

[9] Welter A, Sundararaman S, Li R, Zhang T, Karulin AY, Lehmann A, Naeem V, Roen DR, Kuerten S, Lehmann PV. High-Throughput GLP-Capable Target Cell Visualization Assay for Measuring Cell-Mediated Cytotoxicity. Cells. 2018; 7:E35. 10.3390/cells7050035 29695103 PMC5981259

[10] Zhou J, Hu M, Li J, Liu Y, Luo J, Zhang L, Lu X, Zuo D, Chen Z. Mannan-Binding Lectin Regulates Inflammatory Cytokine Production, Proliferation, and Cytotoxicity of Human Peripheral Natural Killer Cells. Mediators Inflamm. 2019; 2019:6738286. 10.1155/2019/6738286 31915415 PMC6930792

[11] Hou H, Zhou Y, Yu J, Mao L, Bosco MJ, Wang J, Lu Y, Mao L, Wu X, Wang F, Sun Z. Establishment of the Reference Intervals of Lymphocyte Function in Healthy Adults Based on IFN-γ Secretion Assay upon Phorbol-12-Myristate-13-Acetate/Ionomycin Stimulation. Front Immunol. 2018; 9:172. 10.3389/fimmu.2018.00172 29467761 PMC5808316

[12] Qin L, Jing X, Qiu Z, Cao W, Jiao Y, Routy JP, Li T. Aging of immune system: immune signature from peripheral blood lymphocyte subsets in 1068 healthy adults. Aging (Albany NY). 2016; 8:848–59. 10.18632/aging.100894 26886066 PMC4931839

[13] Ding Y, Zhou L, Xia Y, Wang W, Wang Y, Li L, Qi Z, Zhong L, Sun J, Tang W, Liang F, Xiao H, Qin T, et al. Reference values for peripheral blood lymphocyte subsets of healthy children in China. J Allergy Clin Immunol. 2018; 142:970–973.e8. 10.1016/j.jaci.2018.04.022 29746882

[14] Muggen AF, de Jong M, Wolvers-Tettero IL, Kallemeijn MJ, Teodósio C, Darzentas N, Stadhouders R, IJspeert H, van der Burg M, van IJcken WF, Verhaar JA, Abdulahad WH, Brouwer E, et al. The presence of CLL-associated stereotypic B cell receptors in the normal BCR repertoire from healthy individuals increases with age. Immun Ageing. 2019; 16:22. 10.1186/s12979-019-0163-x 31485252 PMC6714092

[15] Johnson JL, Scholz JL, Marshak-Rothstein A, Cancro MP. Molecular pattern recognition in peripheral B cell tolerance: lessons from age-associated B cells. Curr Opin Immunol. 2019; 61:33–38. 10.1016/j.coi.2019.07.008 31446338

[16] Blanco E, Pérez-Andrés M, Arriba-Méndez S, Contreras-Sanfeliciano T, Criado I, Pelak O, Serra-Caetano A, Romero A, Puig N, Remesal A, Torres Canizales J, López-Granados E, Kalina T, Sousa AE, van Zelm M, van der Burg M, van Dongen JJM, Orfao A; EuroFlow PID group. Age-associated distribution of normal B-cell and plasma cell subsets in peripheral blood. J Allergy Clin Immunol. 2018; 141:2208–19.e16. 10.1016/j.jaci.2018.02.017 29505809

[17] Terrén I, Orrantia A, Vitallé J, Zenarruzabeitia O, Borrego F. CFSE dilution to study human T and NK cell proliferation in vitro. Methods Enzymol. 2020; 631:239–55. 10.1016/bs.mie.2019.05.020 31948550

[18] Dons’koi BV, Chernyshov VP, Osypchuk DV. Measurement of NK activity in whole blood by the CD69 up-regulation after co-incubation with K562, comparison with NK cytotoxicity assays and CD107a degranulation assay. J Immunol Methods. 2011; 372:187–95. 10.1016/j.jim.2011.07.016 21839083

[19] Lorenzo-Herrero S, Sordo-Bahamonde C, Gonzalez S, López-Soto A. CD107a Degranulation Assay to Evaluate Immune Cell Antitumor Activity. Methods Mol Biol. 2019; 1884:119–30. 10.1007/978-1-4939-8885-3_7 30465198

[20] Le Garff-Tavernier M, Béziat V, Decocq J, Siguret V, Gandjbakhch F, Pautas E, Debré P, Merle-Beral H, Vieillard V. Human NK cells display major phenotypic and functional changes over the life span. Aging Cell. 2010; 9:527–35. 10.1111/j.1474-9726.2010.00584.x 20477761

[21] Zakiryanova GK, Kustova E, Urazalieva NT, Baimuchametov ET, Nakisbekov NN, Shurin MR. Abnormal Expression of c-Myc Oncogene in NK Cells in Patients with Cancer. Int J Mol Sci. 2019; 20:E756. 10.3390/ijms20030756 30754645 PMC6387292

[22] Sun JC, Lanier LL. Is There Natural Killer Cell Memory and Can It Be Harnessed by Vaccination? NK Cell Memory and Immunization Strategies against Infectious Diseases and Cancer. Cold Spring Harb Perspect Biol. 2018; 10:a029538. 10.1101/cshperspect.a029538 29254979 PMC6005716

[23] Peng H, Tian Z. Natural Killer Cell Memory: progress and Implications. Front Immunol. 2017; 8:1143. 10.3389/fimmu.2017.01143 28955346 PMC5601391

[24] Lukas Yani S, Keller M, Melzer FL, Weinberger B, Pangrazzi L, Sopper S, Trieb K, Lobina M, Orrù V, Fiorillo E, Cucca F, Grubeck-Loebenstein B. CD8^+^HLADR^+^ Regulatory T Cells Change With Aging: They Increase in Number, but Lose Checkpoint Inhibitory Molecules and Suppressive Function. Front Immunol. 2018; 9:1201. 10.3389/fimmu.2018.01201 29915580 PMC5994398

[25] Lins L, Farias É, Brites-Alves C, Torres A, Netto EM, Brites C. Increased expression of CD38 and HLADR in HIV-infected patients with oral lesion. J Med Virol. 2017; 89:1782–87. 10.1002/jmv.24852 28500735

[26] Vallejo AN. CD28 extinction in human T cells: altered functions and the program of T-cell senescence. Immunol Rev. 2005; 205:158–69. 10.1111/j.0105-2896.2005.00256.x 15882352

[27] Hang J, Huang J, Zhou S, Wu L, Zhu Y, Zhu L, Zhou H, Xu K, Jiang H, Yang X. The clinical implication of CD45RA^+^ naïve T cells and CD45RO^+^ memory T cells in advanced pancreatic cancer: a proxy for tumor biology and outcome prediction. Cancer Med. 2019; 8:1326–35. 10.1002/cam4.1988 30767430 PMC6434335

[28] Waaijer ME, Goldeck D, Gunn DA, van Heemst D, Westendorp RG, Pawelec G, Maier AB. Are skin senescence and immunosenescence linked within individuals? Aging Cell. 2019; 18:e12956. 10.1111/acel.12956 31062498 PMC6612632

[29] Rozanski CH, Arens R, Carlson LM, Nair J, Boise LH, Chanan-Khan AA, Schoenberger SP, Lee KP. Sustained antibody responses depend on CD28 function in bone marrow-resident plasma cells. J Exp Med. 2011; 208:1435–46. 10.1084/jem.20110040 21690252 PMC3135367

[30] von Kutzleben S, Pryce G, Giovannoni G, Baker D. Depletion of CD52-positive cells inhibits the development of central nervous system autoimmune disease, but deletes an immune-tolerance promoting CD8 T-cell population. Implications for secondary autoimmunity of alemtuzumab in multiple sclerosis. Immunology. 2017; 150:444–55. 10.1111/imm.12696 27925187 PMC5343359

[31] Chandwani J, Vyas N, Hooja S, Sharma B, Maheshwari R. Mycological Profile of Sputum of HIV Positive Patients with Lower Respiratory Tract Infection and its Correlation with CD4+ T Lymphocyte Count. J Clin Diagn Res. 2016; 10:DC28–31. 10.7860/JCDR/2016/21601.8585 27790435 PMC5071935

[32] Mohd Nor F, Tan LH, Na SL, Ng KP. Meningitis Caused by Rhodotorula mucilaginosa in HIV-Infected Patient: A Case Report and Review of the Literature. Mycopathologia. 2015; 180:95–98. 10.1007/s11046-015-9879-0 25739670

[33] Telles JP, de Andrade Perez M, Marcusso R, Correa K, Teixeira RF, Tobias WM. Hemophagocytic syndrome in patients living with HIV: a retrospective study. Ann Hematol. 2019; 98:67–72. 10.1007/s00277-018-3500-9 30255313

[34] Hazra I, Sk Md OF, Datta A, Mondal S, Moitra S, Singh MK, Chaudhuri S, Das PK, Basu AK, Dhar I, Basu N, Chaudhuri S. T11TS immunotherapy augments microglial and lymphocyte protective immune responses against Cryptococcus neoformans in the brain. Scand J Immunol. 2019; 89:e12733. 10.1111/sji.12733 30450625

[35] Avino LJ, Naylor SM, Roecker AM. Pneumocystis jirovecii Pneumonia in the Non-HIV-Infected Population. Ann Pharmacother. 2016; 50:673–79. 10.1177/1060028016650107 27242349

[36] Oltolini C, Ripa M, Andolina A, Brioschi E, Cilla M, Petrella G, Gregorc V, Castiglioni B, Tassan Din C, Scarpellini P. Invasive Pulmonary Aspergillosis Complicated by Carbapenem-Resistant Pseudomonas aeruginosa Infection During Pembrolizumab Immunotherapy for Metastatic Lung Adenocarcinoma: Case Report and Review of the Literature. Mycopathologia. 2019; 184:181–85. 10.1007/s11046-018-0291-4 30101407

[37] Castelblanco E, Hernández M, Castelblanco A, Gratacòs M, Esquerda A, Molló À, Ramírez-Morros A, Real J, Franch-Nadal J, Fernández-Real JM, Mauricio D. Low-grade Inflammatory Marker Profile May Help to Differentiate Patients With LADA, Classic Adult-Onset Type 1 Diabetes, and Type 2 Diabetes. Diabetes Care. 2018; 41:862–68. 10.2337/dc17-1662 29358494

[38] Qiu X, Song Y, Cui Y, Liu Y. Increased neutrophil-lymphocyte ratio independently predicts poor survival in non-metastatic triple-negative breast cancer patients. IUBMB Life. 2018; 70:529–35. 10.1002/iub.1745 29707892

[39] Qiu J, Zhou F, Li X, Zhang S, Chen Z, Xu Z, Lu G, Zhu Z, Ding N, Lou J, Ye Z, Qian Q. Changes and Clinical Significance of Detailed Peripheral Lymphocyte Subsets in Evaluating the Immunity for Cancer Patients. Cancer Manag Res. 2020; 12:209–19. 10.2147/CMAR.S221586 32021437 PMC6957005

[40] Vincent JL, Moreno R, Takala J, Willatts S, De Mendonça A, Bruining H, Reinhart CK, Suter PM, Thijs LG. The SOFA (Sepsis-related Organ Failure Assessment) score to describe organ dysfunction/failure. On behalf of the Working Group on Sepsis-Related Problems of the European Society of Intensive Care Medicine. Intensive Care Med. 1996; 22:707–10. 10.1007/BF01709751 8844239

[41] Schlapbach LJ, Straney L, Bellomo R, MacLaren G, Pilcher D. Prognostic accuracy of age-adapted SOFA, SIRS, PELOD-2, and qSOFA for in-hospital mortality among children with suspected infection admitted to the intensive care unit. Intensive Care Med. 2018; 44:179–88. 10.1007/s00134-017-5021-8 29256116 PMC5816088

