# Correction for: Establishing immune scoring model based on combination of the number, function, and phenotype of lymphocytes

**DOI:** 10.18632/aging.203423

**Published:** 2021-08-11

**Authors:** Guoxing Tang, Xu Yuan, Ying Luo, Qun Lin, Zhishui Chen, Xue Xing, Huijuan Song, Shiji Wu, Hongyan Hou, Jing Yu, Liyan Mao, Weiyong Liu, Feng Wang, Ziyong Sun

**Affiliations:** 1 Department of Laboratory Medicine, Tongji Hospital, Tongji Medical College, Huazhong University of Science and Technology, Wuhan, China; 2 Institute of Organ Transplantation, Tongji Hospital, Tongji Medical College, Huazhong University of Science and Technology, Wuhan, China; 3 Key Laboratory of Organ Transplantation, Ministry of Education, Ministry of Public Health, Chinese Academy of Medical Sciences, Beijing, China; 4 Department of Nephrology, Tongji Hospital, Tongji Medical College, Huazhong University of Science and Technology, Wuhan, China; 5 The Third Affiliated Hospital of Zhengzhou University, Zhengzhou, China; 6 Center for Cellular and Molecular Diagnosis, Biochemistry and Molecular Biology, Tulane University School of Medicine, New Orleans, LA 70112, USA

**Keywords:** correction

Original article: Aging. 2020; 12:9328–9343.  . https://doi.org/10.18632/aging.103208

**This article has been corrected:** The authors requested to correct the unit of cell number in Table 1 and Table 2 to **μl** instead of ml. This mistake was due to the font corruption. The authors declare that these corrections do not change the results or conclusions of this work.

The corrected Tables are posted below.

**Table 1:** Reference ranges of lymphocyte number, function, and phenotype in different age groups.

**Parameters**		**All**	**Children**	**Adolescents**	**Adults**	**Elders**	**p**
		**N=261**	**N=47**	**N=72**	**N=90**	**N=52**	
Age	Mean±SD (Range)	33.68±26.63 (1-82)	3.81±1.04 (1-5)	10.81±3.74 (6-18)	46±13.79 (18-65)	71.04±4.39 (66-82)	
Sex	Male:Female	168:93	35:12	52:20	53:37	28:24	
**Number**							
CD4^+^ T cell number (/μl)	Mean±SD (2.5%-97.5%)	836±355 (374-1881)	1260±399 (635-1979)	927±287 (560-1653)	648±203 (360-1074)	652±187 (367-1007)	<0.001
CD8^+^T cell number (/μl)	Mean±SD (2.5%-97.5%)	576±310 (154-1459)	880±343 (426-1553)	728±262 (397-1382)	424±171 (180-847)	355±154 (116-681)	<0.001
B cell number (/μl)	Mean±SD (2.5%-97.5%)	358±252 (73-1006)	695±251 (251-1240)	457±173 (218-905)	184±81 (53-352)	216±140 (67-537)	<0.001
NK cell number (/μl)	Mean±SD (2.5%-97.5%)	383±219 (103-920)	381±243 (89-905)	345±228 (86-934)	364±165 (154-732)	473±239 (125-1000)	<0.05
**Function**							
IFN-g^+^CD4^+^ T cells (%)	Mean±SD (2.5%-97.5%)	17.84±8.85 (6.62-36.81)	10.23±4.16 (4.08-17.17)	12.28±4.86 (5.43-20.35)	23.72±8.12 (12.34-40.53)	22.26±7.52 (8.83-34.43)	<0.001
IFN-g^+^CD8^+^ T cells (%)	Mean±SD (2.5%-97.5%)	46.25±22.43 (13.59-87.72)	26.89±10.77 (8.95-46.18)	29.13±10.83 (13.63-56.57)	56.08±17.92 (22.76-87.38)	70.46±14.33 (42.84-92.36)	<0.001
IFN-g^+^ NK cells (%)	Mean±SD (2.5%-97.5%)	72.68±12.65 (43.88-90.94)	67.28±14.57 (40.76-85.99)	67.77±13.16 (39.70-87.66)	77.29±9.95 (57.73-91.48)	76.37±9.52 (59.79-90.61)	<0.001
**Phenotype**							
CD28^+^CD4^+^ T cells (%)	Mean±SD (2.5%-97.5%)	94.95±7.03 (73.33-99.97)	98.17±4.03 (89.50-99.98)	97.92±2.72 (88.95-99.95)	92.81±7.6 (71.81-99.88)	91.61±9.10 (66.26-99.83)	<0.001
HLA-DR^+^CD4^+^ T cells (%)	Mean±SD (2.5%-97.5%)	14.33±7.45 (5.42-32.78)	9.68±4.79 (5.29-24.40)	10.64±4.15 (5.11-19.71)	16.23±7.02 (5.97-34.34)	20.35±8.25 (9.07-40.22)	<0.001
CD45RO+CD4^+^T cells (%)	Mean±SD (2.5%-97.5%)	50.89±18.88 (20.58-88.47)	28.77±7.71 (13.63-43.39)	40.37±9.88 (22.48-58.08)	61.09±14 (36.21-88.06)	67.82±14.07 (37.46-93.85)	<0.001
CD28^+^CD8^+^ T cells (%)	Mean±SD (2.5%-97.5%)	62.06±17.3 (26.41-88.91)	71.58±12.49 (52.47-91.71)	71.39±12.36 (45.28-88.65)	58±15.87 (26.72-84.13)	47.57±16.5 (20.60-82.40)	<0.001
HLA-DR^+^CD8^+^ T cells (%)	Mean±SD (2.5%-97.5%)	34.93±17.12 (9.97-71.53)	23.26±11.82 (7.42-47.14)	25.13±10.45 (9.98-46.31)	39.09±15.71 (14.96-72.95)	51.83±13.98 (22.97-74.98)	<0.001

SD: standard deviation. *P* means association between different parameters and age in all participants by using Spearman's rank correlation test.

**Table 2:** Reference ranges of calculated parameters in different age groups.

**Parameters**		**All**	**Children**	**Adolescents**	**Adults**	**Elders**	**p**
		**N=261**	**N=47**	**N=72**	**N=90**	**N=52**	
Age	Mean±SD (Range)	33.68±26.63 (1-82)	3.81±1.04 (1-5)	10.81±3.74 (6-18)	46±13.79 (18-65)	71.04±4.39 (66-82)	
CD4^+^ T cell number × function (/μl)	Mean±SD (2.5%-97.5%)	134±66 (56-293)	125±58 (57-225)	108±40 (57-209)	153±75 (61-328)	147±73 (44-311)	<0.001
CD8^+^ T cell number × function (/μl)	Mean±SD (2.5%-97.5%)	237±139 (72-567)	240±143 (65-565)	212±126 (86-489)	242±143 (79-564)	259±74 (67-553)	<0.05
NK cell number × function (/μl)	Mean±SD (2.5%-97.5%)	283±175 (49-736)	254±157 (37-585)	241±184 (44-780)	286±142 (90-674)	216±125 (80-832)	<0.001
CD4^+^ T cell number × CD28^+^CD4^+^ T cells % (/μl)	Mean±SD (2.5%-97.5%)	798±359 (333-1844)	1236±392 (631-1925)	909±288 (536-1638)	600±190 (318-1001)	594±173 (320-894)	<0.001
CD4^+^ T cell number × HLA-DR^+^CD4^+^ T cells % (/μl)	Mean±SD (2.5%-97.5%)	110±60 (36-257)	121±70 (43-299)	95±37 (44-176)	103±52 (35-232)	135±78 (47-339)	<0.01
CD4^+^ T cell number × CD45RO^+^CD4^+^ T cells % (/μl)	Mean±SD (2.5%-97.5%)	385±140 (192-709)	347±109 (220-557)	358±94 (225-570)	393±153 (165-735)	444±168 (219-748)	<0.001
CD8^+^ T cell number × CD28^+^CD8^+^ T cells % (/μl)	Mean±SD (2.5%-97.5%)	364±232 (82-949)	618±233 (243-1011)	508±174 (262-950)	236±98 (104-435)	134±50 (57-286)	<0.001
CD8^+^ T cell number × HLA-DR^+^CD8^+^ T cells % (/μl)	Mean±SD (2.5%-97.5%)	187±129 (41-547)	221±183 (38-565)	185±113 (67-452)	169±110 (39-478)	132±48 (46-436)	>0.05

SD: standard deviation. *P* means association between different parameters and age in all participants by using Spearman's rank correlation test.

