# Supplementary material for: Establishing immune scoring model based on combination of the number, function, and phenotype of lymphocytes
Source: Aging (Albany NY). 2020 May 12;12(10):9328–43. doi: 10.18632/aging.103208 (PMC7288950; doi:10.18632/aging.103208)
Supplement: Supplementary Figure 1 [file aging-12-103208-s001..pdf]

SUPPLEMENTARY FIGURE

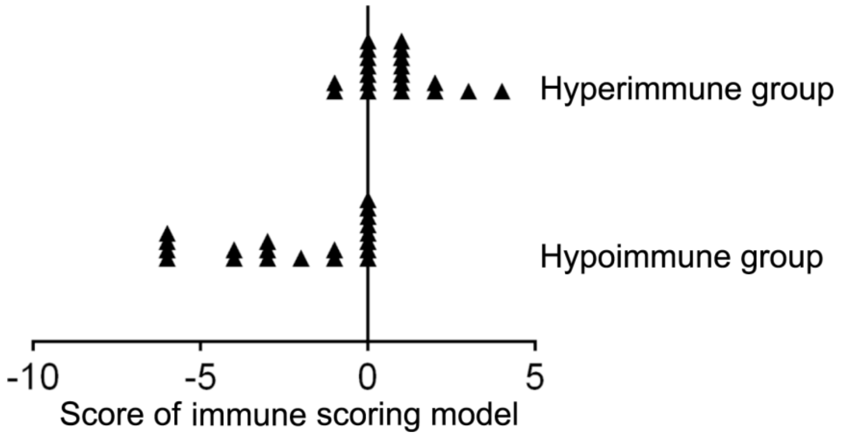

Supplementary Figure 1. The score distribution of the immune scoring model (based on combination of lymphocyte number and function) in patients with hyperimmune and hypoimmune status.
