# Supplementary material for: Establishing immune scoring model based on combination of the number, function, and phenotype of lymphocytes
Source: Aging (Albany NY). 2020 May 12;12(10):9328–43. doi: 10.18632/aging.103208 (PMC7288950; doi:10.18632/aging.103208)
Supplement: Supplementary Tables [file aging-12-103208-s002..pdf]

SUPPLEMENTARY TABLES

Supplementary Table 1. Reference ranges of the percentages of T cells, B cells, and NK cells in different age groups.

Parameters		All<br>N=261	Children<br>N=47	Adolescents<br>N=72	Adults<br>N=90	Elders<br>N=52	p
Age	Mean±SD<br>(Range)	33.68±26.63<br>(1-82)	3.81±1.04<br>(1-5)	10.81±3.74<br>(6-18)	46±13.79<br>(18-65)	71.04±4.39<br>(66-82)	
Sex	Male: Female	168:93	35:12	52:20	53:37	28:24	
T cell number	Mean±SD<br>(2.5%-97.5%)	1559±688<br>(612-3313)	2378±733<br>(1214-3832)	1845±515<br>(1077-3035)	1173±363<br>(681-2021)	1092±314<br>(589-1712)	<0.001
T cells (%)	Mean±SD<br>(2.5%-97.5%)	66.71±7.81<br>(47.74-80.22)	67.68±6.24<br>(56.62-78.24)	69.19±5.5.98<br>(57.84-80.08)	67.62±7.28<br>(51.55-81.59)	60.82±9.18<br>(44.50-76.68)	<0.001
B cells (%)	Mean±SD<br>(2.5%-97.5%)	14.44±6.29<br>(4.5-29.32)	20.45±6.2<br>(10.33-34.30)	17.18±4.69<br>(9.71-26.50)	10.65±3.69<br>(3.83-17.60)	11.76±5.9<br>(4.33-26.47)	<0.001
NK cells (%)	Mean±SD<br>(2.5%-97.5%)	17.93±9.85<br>(3.99-40.72)	10.69±5.95<br>(3.51-21.51)	12.71±6.57<br>(3.92-26.58)	20.9±7.42<br>(9.6-37.97)	26.54±11.22<br>(8.73-47.73)	<0.001
$CD4^+$ T cells (%)	Mean±SD<br>(2.5%-97.5%)	36.39±6.9<br>(23.18-49.33)	36.13±6<br>(25.56-45.85)	34.92±6.18<br>(23.59-49.08)	37.56±7.32<br>(24.82-51.33)	36.61±7.45<br>(22.27-49.13)	<0.05
$CD8^+$ T cells (%)	Mean±SD<br>(2.5%-97.5%)	24.33±6.71<br>(11.72-38.46)	24.77±5.05<br>(16.69-33.93)	27.18±5.55<br>(18.31-38.41)	24.45±6.63<br>(13.88-38.73)	19.78±7.22<br>(8.16-35.13)	<0.001

SD: standard deviation. P means association between different parameters and age in all participants by using Spearman's rank correlation test.

Supplementary Table 2. Reference ranges of lymphocyte number, function, and phenotype in different gender groups.

Parameters		all<br>N=261	Male<br>N=168	Female<br>N=93	p
T cell number	Mean±SD<br>(2.5%-97.5%)	1559±688<br>(729-2946)	1589±670<br>(612-3056)	1505±715<br>(651-3357)	>0.05
T cells (%)	Mean±SD<br>(2.5%-97.5%)	66.71±7.81<br>(51.28-77.92)	66.09±7.92<br>(47.19-78.47)	67.83±7.49<br>(52.36-82.46)	>0.05
$CD4^+$ T cells (%)	Mean±SD<br>(2.5%-97.5%)	36.39±6.9<br>(25.36-47.92)	35.13±6.74<br>(23.68-48.73)	38.65±6.62<br>(23.93-50.45)	<0.001
$CD4^+$ T cell number	Mean±SD<br>(2.5%-97.5%)	836±355<br>(391-1598)	830±339<br>(373-1688)	847±383<br>(379-1892)	>0.05
$CD8^+$ T cells (%)	Mean±SD<br>(2.5%-97.5%)	24.33±6.71<br>(13.85-35.67)	24.81±6.82<br>(11.71-38.94)	23.45±6.41<br>(12.63-35.65)	>0.05
$CD8^+$ T cell number	Mean±SD<br>(2.5%-97.5%)	576±310<br>(190-1132)	604±306<br>(153-1442)	527±310<br>(183-1391)	>0.05
B cells (%)	Mean±SD<br>(2.5%-97.5%)	14.44±6.29<br>(5.94-25.43)	14.65±6.44<br>(4.36-30.69)	14.06±6.00<br>(5.57-27.63)	>0.05
B cell number	Mean±SD<br>(2.5%-97.5%)	358±252<br>(83-867)	369±237<br>(72-908)	337±276<br>(76-1143)	>0.05
NK cells (%)	Mean±SD<br>(2.5%-97.5%)	17.93±9.85<br>(5.16-36.6)	18.32±10.39<br>(4.02-46.07)	17.21±8.76<br>(4.11-33.29)	>0.05
NK cell number	Mean±SD<br>(2.5%-97.5%)	383±219<br>(125-851)	406±229<br>(105-943)	343±191<br>(98-740)	<0.05
IFN-γ^+CD4^+ cells (%)	Mean±SD<br>(2.5%-97.5%)	17.84±8.85<br>(6.27-34.22)	16.74±8.37<br>(5.67-34.98)	19.83±9.35<br>(5.07-38.36)	<0.01
IFN-γ^+CD8^+ T cells (%)	Mean±SD<br>(2.5%-97.5%)	46.25±22.43<br>(14.64-85.51)	43.76±22.17<br>(13.57-87.56)	50.76±22.20<br>(13.75-87.72)	<0.05
IFN-γ^+NK cells (%)	Mean±SD<br>(2.5%-97.5%)	72.68±12.65<br>(49.96-89.57)	72.57±12.26<br>(44.91-90.71)	72.87±13.34<br>(42.97-90.87)	>0.05
HLA-DR^+ T cells (%)	Mean±SD<br>(2.5%-97.5%)	16.23±7.54<br>(6.82-31.29)	16.24±7.69<br>(6.06-34.44)	16.21±7.24<br>(6.54-33.06)	>0.05
$CD28^+CD4^+$ T cells(%)	Mean±SD<br>(2.5%-97.5%)	94.95±7.03<br>(75.97-99.92)	95.22±7.07<br>(72.25-99.97)	94.44±6.94<br>(73.95-99.94)	>0.05
HLA-DR^+CD4^+ T cells (%)	Mean±SD<br>(2.5%-97.5%)	14.33±7.45<br>(5.96-29.07)	14.2±7.42<br>(5.87-30.90)	14.57±7.50<br>(4.89-32.89)	>0.05
$CD45RO^+CD4^+$ T cells (%)	Mean±SD<br>(2.5%-97.5%)	50.89±18.88<br>(23.29-82.03)	49.53±18.78<br>(20.13-83.54)	53.35±18.80<br>(21.59-84.33)	>0.05
$CD45RA^+CD4^+$ T cells (%)	Mean±SD<br>(2.5%-97.5%)	49.04±18.88<br>(11.53-79.42)	50.43±18.75<br>(11.76-79.87)	46.55±18.87<br>(15.67-78.42)	>0.05
$CD28^+CD8^+$ T cells (%)	Mean±SD<br>(2.5%-97.5%)	62.06±17.3<br>(29.46-86.08)	62.61±17.78<br>(26.39-89.32)	61.08±16.34<br>(27.84-88.06)	>0.05
HLA-DR^+CD8^+ T cells (%)	Mean±SD<br>(2.5%-97.5%)	34.93±17.12<br>(12.47-66.36)	34.75±17.24<br>(9.89-72.99)	35.24±16.90<br>(11.30-68.72)	>0.05

SD: standard deviation. P means association between different parameters and age in all participants by using Spearman's rank correlation test.

Supplementary Table 3. The demographic and clinical characteristics of patients.

**Characteristic**	**Hypoimmune group (n=21)**	**Hyperimmune group (n=20)**
Mean age (mean±SD), years	49±16	58±11
Male: Famale	16:5	13:7
Underlying diseases		
malignancy undergoing therapy	8(38.09)	
autoimmune disease receiving treatment	3(14.29)	
transplantation receiving treatment	2(9.52)	
diabetes more than 10 years	3(14.29)	
chronic renal failure	1(4.76)	

SD: standard deviation.
